# Emerging Pharmaceutical Therapies to Address the Inadequacy of a Gluten-Free Diet for Celiac Disease

**DOI:** 10.3390/ph17010004

**Published:** 2023-12-20

**Authors:** Martina Crepaldi, Michela Palo, Daria Maniero, Luisa Bertin, Edoardo Vincenzo Savarino, Robert P. Anderson, Fabiana Zingone

**Affiliations:** 1 Department of Surgery, Oncology and Gastroenterology, University of Padua, 35128 Padua, Italy; martina.crepaldi.3@studenti.unipd.it (M.C.); michela.palo@unipd.it (M.P.); daria.maniero@unipd.it (D.M.); luisa.bertin.1@studenti.unipd.it (L.B.); edoardo.savarino@unipd.it (E.V.S.); 2 Gastroenterology Unit, Azienda Ospedale—Università Padova, 35128 Padua, Italy; 3 Gastroenterology Department, Mackay Base Hospital, Mackay, QLD 4740, Australia

**Keywords:** celiac disease (CeD), gluten-free diet (GFD), novel treatment, non-dietary treatment

## Abstract

Celiac disease (CeD) is a chronic autoimmune disorder triggered by the ingestion of gluten, affecting around 1% of the global population. It is a multifactorial disease involving both genetics and environmental factors. Nowadays, the only available treatment for CeD is a life-long gluten-free diet (GFD), which can cause a significant burden for patients, since symptoms and mucosal injury can persist despite apparent compliance with a GFD. This could also lead to psychological consequences and affect the quality of life of these patients. Thankfully, recent advances in understanding the pathogenesis of CeD and the availability of various targets have made it feasible to explore pharmaceutical treatments specific to CeD. Recently, the FDA has highlighted the unmet needs of adult patients on a GFD who experience ongoing symptoms attributed to CeD and also show persistent duodenal villous atrophy. This review will outline the limitations of a GFD, describe the targets of potential novel treatment of CeD and provide an overview of the primary clinical trials involving oral and injectable agents for a non-dietary treatment of CeD.

## 1. Introduction

Celiac disease (CeD) is defined by chronic, gluten-dependent small bowel enteropathy associated with autoantibody directed against transglutaminase 2 (TG2) and an acquired CD4+ T cell-driven immune response directed against dietary gluten. The global prevalence of CeD based on serology is around 1.4% or 0.7% if limited to only those confirmed by histology. Females are affected more often than males, although the exact ratio varies from study to study. CeD is furthermore more often diagnosed in adults but usually develops in infancy [1,2,3]. CeD is not curable, but enteropathy usually improves the condition sufficiently with a gluten-free diet (GFD) to reverse chronic symptoms and malabsorption. Recent data based on quantitative histomorphometries [4], however, have questioned the conventional understanding that a strict GFD leads to complete histological remission in most patients after a year, and almost all patients after three/five years on a GFD [5,6,7,8]. Indeed, Marsh 2 lesions are seldom reported in follow-up studies [9,10], but are present in one-third or more CeD patients on a GFD when quantitative histomorphometry is deployed [11]. Using conventional subjective histology, Silva et al. observed histological remission, defined as Marsh 0, in about one-third of their patients [8], which contrasts with Daveson et al. who, using a validated quantitative histomorphometry protocol, reported 8% or less of adults with well-controlled CeD on a GFD had Marsh 0 or 1 [11].

Growing concern that a GFD alone is often insufficient to reverse gluten-induced enteropathy and advancing the understanding of the immunological basis for CeD have stimulated a global effort to develop pharmaceuticals therapies as adjuncts to a GFD. The purpose of this review is to outline the limitations of a GFD, describe the targets of potential novel treatment of CeD and provide an overview of the primary clinical trials involving oral and injectable agents for a non-dietary treatment of CeD.

## 2. Pathogenesis

### 2.1. Genetics

Celiac disease is a multifactorial disease involving both genetics and environmental factors [12]. Genetical susceptibility to CeD is conveyed by genes facilitating a potent adaptive immune response directed against deamidated gluten peptides (DGPs), which include at least one copy of both major histocompatibility complex class II (MHC-class II) genes encoding HLA-DQ2.5 in about 90% of patients or otherwise HLA-DQ8, DQ2.2 or DQ7 [13]. The exceptionally strong association between CeD and MHC-class II genes contrasts with IgE-associated and other non-IgE-associated food allergies, and also highlights that wheat allergy is a different condition from CeD [14,15].

The MHC-class II genes are indeed the driving genetic factor for CeD, but there are numerous non-MHC genes that can increase the risk for CeD, which are primarily located in non-coding regions [16,17]. The MHC-class II genes coding susceptible HLA-DQ polymorphisms are necessary but not sufficient for the development of the disease; their absence is helpful to exclude the diagnosis of CeD in case of equivocal small bowel histological findings [18]. 

### 2.2. Gluten Digestion and Absorption

The critical environmental factor essential for the development of CeD is enteric exposure to gluten proteins derived from wheat, barley, rye and, for some CeD patients, also those in oats [19,20]. Wheat gliadins and the related hordeins in barley and secalins in rye are the most potent proteins reactivating the adaptive immune response to gluten in patients with CeD [21]. Regions of these proteins rich in proline and glutamine are relatively resistant to human brush border and pancreatic endopeptidases. Consequently, intact gliadin peptides sufficiently large to be immunogenic are relatively abundant and available for paracellular or immunoglobulin A (IgA)-facilitated transcellular transport across the intestinal epithelium into the lamina propria. 

### 2.3. Deamidation and Immune Recognition of Gluten Peptides

These relatively large gliadin peptides acquire immunogenicity following partial deamidation occurring before or during the course of absorption. Transglutaminase 2 (TG2), formerly known as tissue transglutaminase, expressed in apoptotic and injured cells in the gut has been implicated as the host enzyme responsible for facilitating gluten immunotoxicity in celiac disease. TG2 selectively deamidates gliadin peptides, yielding two distinct products that either activate CD4+ T cells via the T cell receptor (soluble deamidated gliadin peptide, DGP) or bind soluble B-cell surface-bound immunoglobulin (the B cell receptor). TG2 in acidic conditions catalyzes the direct deamidation of glutamine incorporated in peptide to glutamate, producing DGPs. Alternatively, TG2 in neutral and alkaline conditions catalyzes transamidation, linking peptide glutamine to amines such as lysine residues in proteins that allows for the formation of deamidated gliadin–TG2 complexes that function as highly immunogenic hapten-carrier complexes driving both DGP-specific CD4+ T cells and B cells specific for DGP or TG2. Detailed crystallography studies have defined the structural interactions between HLA-DQ2.5, HLA-DQ8 and immunogenic gluten peptides [22,23], and also with cognate T cell receptor [24]. CD4+ T cell recognition of DGPs is facilitated firstly because selective deamidation substantially increases the binding avidity of many gliadin peptides for HLA-DQ2.5 and/or HLA-DQ8 preferentially expressed on the surface of professional antigen-presenting cells (dendritic cells and B cells). CD4+ T cell recognition of DGPs is also facilitated by DGP-TG2 complexes because specific B cells (for DGP and TG2) are exceptionally efficient antigen-presenting cells, allowing immune recognition of tiny amounts of DGPs that would be impossible with dendritic cells or macrophages alone. 

In summary, gut digestive proteases and TG2, along with facilitated transepithelial transport, are understood as having key roles in delivering sufficient gluten antigen to allow dendritic cells activated by innate stimuli to drive maturation of naive DGP-specific CD4+ T cells (disease induction), and also for DGP- and TG2-specific B cells to drive highly efficient reactivation and expansion of memory DGP-specific CD4+ T cells in established disease. Cytokine and chemokine release by activated DGP-specific CD4+ T cells directly injures epithelial cells, recruits and activates innate immune cells to amplify intestinal injury, expands the population of DGP-specific CD4+ cells and TG2- and DGP-specific B cells, and also drives B cell maturation to produce plasma cells secreting IgA and IgG specific for TG2 and DGP.

### 2.4. Gut and Systemic Effects of Activated Gluten Immunity

The cooperative interaction between IL-15 (produced by injured intestinal epithelial cells) and cytokines (derived from the activation of CD4+ T cells and innate immune cells) leads to the differentiation of intraepithelial lymphocytes (IELs) into cytotoxic CD8+ T cells [25,26,27]. One of the pathways activated by these cytokines is the Janus kinase 1-signal transducer and activator of the transcription 3 (JAK1-STAT3) pathway. The inflammatory cascade and cytotoxic CD8+ T cells damage the intestinal mucosa and cause apoptosis. In the presence of proinflammatory cytokines, there is also a low concentration of TGFβ, which promotes CD4+ T helper 17 (Th17) differentiation. Gliadin-specific Th17 CD4+ T cells produce proinflammatory cytokines and mucosa-protective cytokine IL-22 and are an effector memory cell [28]. In established CeD, gluten ingestion drives cytokine (interleukin-2, IL-2) release from specific CD4+ T cells that can be measured in the plasma within two hours and correlates with severity and onset of acute symptoms [29,30,31,32]. In contrast to the rapid effects of gluten on T cell activation, sustained gluten exposure is necessary to induce histological injury in the duodenum. A randomized trial found that 10 g of gluten (more than 3 g) for 14 days could be used as a gluten challenge [33]. Recently, Singh et al. concluded that in children, a minimum of 3–6 g of gluten per day for over 12 weeks was necessary to optimize diagnostic accuracy for evaluation of CeD [34]. 

### 2.5. Microbiome Disturbance in Celiac Disease 

The gut microbiota plays a further role in the development of CeD with different possible mechanisms, for example, the expression of epitopes that mimic gliadin, the overgrowth of certain bacteria associated with increased intestinal permeability, or the activation of the innate and adaptive immune system by lipopolysaccharides [35]. At CeD diagnosis, dysbiosis in fecal and duodenal specimens is characterized by higher numbers of Gram-negative bacteria (*Bacteroides* and *Enterobacteriaceae*) and decreases in the number of beneficial Gram-positive bacteria (*Bifidobacterium* spp.) in comparison to healthy individuals [36,37,38,39].

## 3. Gluten-Free Diet

The only available “specific” treatment for CeD is life-long removal of the causative antigen, i.e., a gluten-free diet (GFD). This therapy has been known since the 1940s, when Willem Dicke identified wheat intake as a cause for reactivation of CeD (Winter Starvation 1944–1945). Strict adherence to a GFD is associated with reductions in serum levels and coeliac-related TG2 IgA and DGP IgG, complete or more often partial recovery of the intestinal mucosa, resolution of iron and nutritional deficiencies and reductions in long-term complications [40]. 

**Figure 1:**
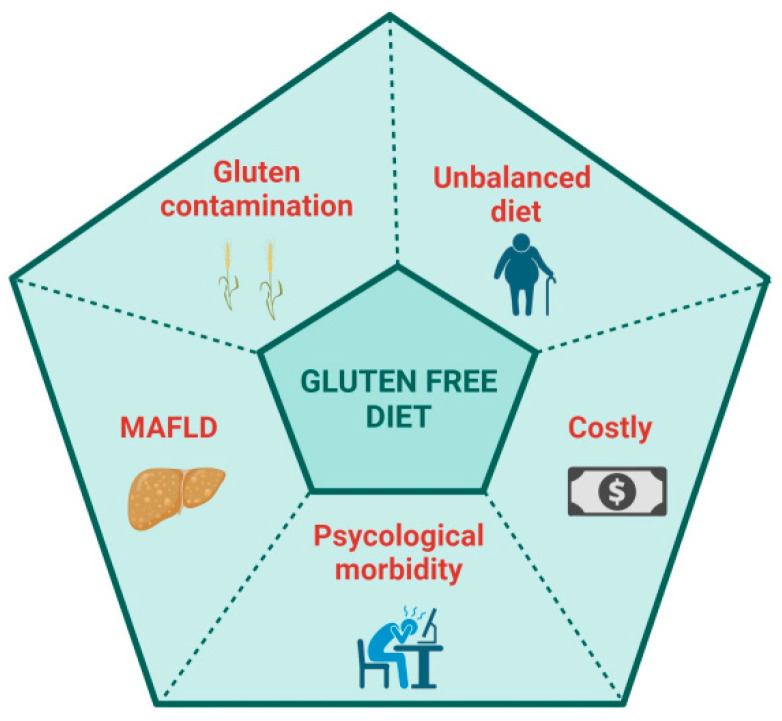
Possible disadvantages of a gluten-free diet. Created with BioRender.com.

A GFD is palliation rather than a cure because the adaptive immune response to deamidated gluten persists, and, in fact, many patients on a GFD report being more symptomatic following gluten ingestion than when they regularly consumed gluten. Indeed, DGP-specific memory CD4+ T cells circulate in the blood of CeD patients at frequencies only modestly lower than in patients with untreated CeD [41]. Patients on a GFD are sometimes susceptible to severe acute gluten-induced toxicity associated with systemic cytokine release [30,32]. A GFD in most patients achieves a substantial quantitative reduction in known ingestion of gluten. A GFD, however, does not eliminate inadvertent gluten exposure resulting from cross-contamination, incorrect or misinterpreted food labelling or ignorance of food vendors and providers unknowingly serving food containing gluten. A GFD rarely achieves exclusion of all dietary gluten, evidenced by persistent mucosal injury in most patients who appear to be well controlled on a GFD [11]. Despite the benefits that a GFD can give, many patients are not satisfied with a GFD and desire a novel, proactive therapy which could control gluten-associated symptoms, reduce the burden of a GFD and improve their quality of life (QoL) [42,43,44] (Figure 1). Physicians and regulators on the other hand are focused on persistent tissue injury and seek treatments that improve objective assessments of intestinal damage such as villous atrophy [45].

Many patients fear gluten contamination. Efforts to study dose-dependent effects of chronic ingestion of a small amount of gliadin on duodenal histology began over 30 years ago [46]. Assessment of histological deterioration is challenging but appeared to be more pronounced in children ingesting 300 mg of gluten daily than those with a daily gluten dose of 100 mg. Subsequently, Catassi et al. performed a prospective, randomized, double-blind, placebo-controlled study with patients receiving a placebo or 10 mg or 50 mg of gluten for 3 months. In this highly cited study, Catassi et al. concluded that ingesting 50 mg gluten daily for 3 months caused mucosal injury in the second part of the duodenum, assessed by a significant decrease in the villous height (Vh) to crypt depth (Cd) ratio, Vh:Cd [47]. A systematic review in 2008 concluded that a daily gluten intake of <10 mg is unlikely to cause significant abnormalities detectable by conventional histology [48]. Unintentional ongoing gluten exposure is more common than commonly believed, even when patients self-report excellent/good GFD adherence. Traces of gluten contamination could be a contributing factor to persistent villous atrophy and the lack of clinical recovery [49,50]. 

Whether weeks or months long, gluten challenge with 10 mg or 50 mg is a valid method to quantitate the upper level of gluten tolerated by patients and has been brought into question by more recent studies measuring fecal gluten immunogenic peptides (GIPs), showing many patients on a GFD often inadvertently ingest about 150 mg [51]. The presence of GIPs in urine and stool reveals transgressions in the gluten-free diet and indirectly incomplete mucosal healing. Single GIP determination could detect daily ingestions of 50 mg of gluten in 15–50% of patients, and 97–100% of patients with an unrestricted gluten intake (>5 g) [52,53,54,55,56,57]. The use of several fecal and/or urine samples at different days and times of the day significantly improves the sensitivity and accuracy of the assessment of diet compliance of these patients.

In addition, rigorous validation of Vh:Cd, or of Vh or Cd alone, in adjacent villus-crypt units has led to an appreciation that very few CeD patients on a GFD achieve a “normal” Vh:Cd, with about half having persistent villous atrophy [58]. As a potentially more sensitive alternative to the second part of the duodenum histology after an extended gluten challenge, systemic IL-2 release within hours after gluten ingestion may be a more sensitive and flexible readout of gluten toxicity [29,32]. The results of an ongoing study utilizing double-blind single bolus gluten challenge with assessments of serum IL-2 to determine the lower level of gluten toxicity are awaited with interest (ACTRN12621000781842).

The risk of contamination and the difficulty of diet adherence make the GFD a restrictive diet, affecting the patient’s quality of life (QoL) and impacting social events, relationships, and work. The previous literature has paid attention to the QoL of CeD patients, both at diagnosis and while on a GFD, mainly finding a reduction in QoL at diagnosis, which improves once on a GFD [59]. Nachman et al. observed that QoL and depression scales were significantly worse at 4 years post-diagnosis compared with 1 year, even if the scores remained significantly better than those at diagnosis [60], finding that low adherence to a GFD mainly impacted QoL. A year later, Barratt et al. found that the perceived degree of difficulty of adhering to a GFD impacted QoL [61]. In a systematic review by Burger et al., the authors assessed that a GFD significantly improves but does not normalize health-related quality of life, confirming that better dietary adherence results in a higher QoL [62]. Marsilio et al. found that non-compliant GFD patients appeared to suffer from dysphoria, a generalized dissatisfaction with life [63]. A prospective study has recently reported an improvement in QoL and psychological disorders after one and two years on a GFD, describing dietary compliance as the leading risk factor [64,65]. Even when the GFD is well done, another point recently underlined is the risk of developing metabolic syndrome and a fatty liver [66,67]. Rispo et al. have recently described an increased risk of metabolic-associated fatty liver disease (MAFLD) in CeD patients on a GFD [68]. The authors found that 46.6% of patients developed NAFLD at two years of follow-up, while 32.6% had MAFLD. However, other studies have found contrasting results [69,70,71]. 

Finally, the literature describes that 7–30% of CeD patients are defined as slow responders, since they continue to have symptoms or signs of CeD or laboratory abnormalities after at least 6–12 months of a GFD [72]. In these cases, a differential diagnosis is required, and it is based firstly on a review of the original diagnosis; the available results of biopsy at the diagnosis, serology and/or HLA-DQ2/DQ8 should be analyzed. If CeD is confirmed, the unintentional ingestion of gluten is the most common cause of slow-responder patients (35–50% of cases). Dietary compliance should be the keystone of further evaluations (for example, detecting immunogenic gluten peptides in stool or urine) [56]. CeD serology could also be helpful, but it must be considered that routine serology does not exclude low-level gluten ingestion [73]. Small bowel histology of new biopsy samples can lead to s differential diagnosis once the dietary etiology is excluded. The possible causes for persistent symptoms include irritable bowel syndrome, microscopic colitis, lactose intolerances, bile acid diarrhea and only rarely refractory CeD [72]. In conclusion, a life-long GFD is the only supportive care now available for management of CeD and presents a significant burden for patients; however, strict adherence seldom completely eliminates dietary gluten and symptoms and mucosal injury can persist despite apparent compliance with the GFD. Fortunately, CeD-specific pharmaceutical development is now possible because CeD immuno-pathogenesis is now relatively well understood, and a variety of biomarkers are available to assess therapeutic efficacy. 

## 4. Pharmaceutical Adjuncts to a GFD

Indications for pharmaceutical agents are likely to be as adjunct rather than replacements for a GFD [45]. Many CeD patients already have access to “over-the-counter” supplements that claim to provide some relief or protection from the effects of gluten; however, there is no pharmaceutical agent that has yet received approval from regulatory agencies for the “treatment” of gastrointestinal effects of CeD. Dapsone, however, is approved by the United States Food and Drug Administration (FDA) for the treatment of dermatitis herpetiformis, a classical but uncommon skin manifestation of CeD [74]. The 2022 draft regulatory guidance from the FDA highlights the unmet needs of adult patients on a GFD who experience ongoing symptoms attributed to CeD and also show persistent duodenal villous atrophy [45]. In addition to national regulatory approvals, availability of drugs for CeD patients would also be shaped by payers, including government agencies seeking cost-effective as well as efficacious medications. This would be a notable change, as patients and families currently bear most or all the cost of treatment of a GFD, and for many patients, the role of the physician in disease management is minimal after a diagnosis is made. 

The FDA statement anticipates drug approvals for CeD that would be prescribed by physicians and foreshadows unpreceded changes in the clinical management of CeD. Presumably, in the future, gastroenterologists will serve as key players in selecting CeD patients likely to benefit from new therapies. This may present a significant challenge since many patients are not followed after diagnosis in specialist clinics, and few have regular follow-up endoscopies to assess mucosal health, even though reports linked to sponsored clinical trials suggest at least half of patients apparently well controlled on a GFD have persistent villous atrophy (Marsh 3) and most of the remainder have crypt hyperplasia (Marsh 2) [5,11]. 

As clinical care of CeD enters the pharmaceutical era, capsule endoscopy to assess the anatomical extent of intestinal involvement and blood tests capable of detecting CeD-associated gluten immunity may become useful clinical tools. Blood tests measuring circulating gluten-specific CD4+ T cells or measuring IL-2 in the serum after bolus gluten challenge could confirm CeD diagnosis and possibly also stratify disease severity based on the “strength” of the immune response to gluten in vivo [32,75,76]. A further challenge is misdiagnosis of CeD amongst patients on a GFD, which may be rather common in patients who have normalized histology [77]. In contrast to a negative CeD-serology result in a patient on a GFD, a negative blood test measuring gluten-specific T cell immunity may allow resumption of an unrestricted diet in many patients strictly avoiding gluten [77].

## 5. Delivery and Administration of Pharmaceutical Adjuncts to a GFD

Investigational products under development for CeD are delivered either orally or by subcutaneous or intravenous routes. Oral medications may require strictly timed administration before meals to be effective. For example, unless the possibility of gluten exposure can be anticipated, glutenases digesting gluten contaminants may be needed before every meal or snack. Potentially, the same rigorous compliance may also be needed for oral inhibitors of TG2 if their duration of action is short. In contrast, injectables that aim to desensitize or tolerize the immune system to gluten would be expected to act over weeks or months, allowing a greater flexibility in the frequency of dosing, but would impose the responsibility of self-injections or presenting to hospital- or community-based infusion clinics on patients. In either scenario, gastroenterologists would be expected to monitor effectiveness and guide selections of alternative medications in case of treatment failure.

## 6. Potential Therapeutic Targets in Celiac Disease

### 6.1. Oral Agents

#### 6.1.1. Steroids

**Figure 2:**
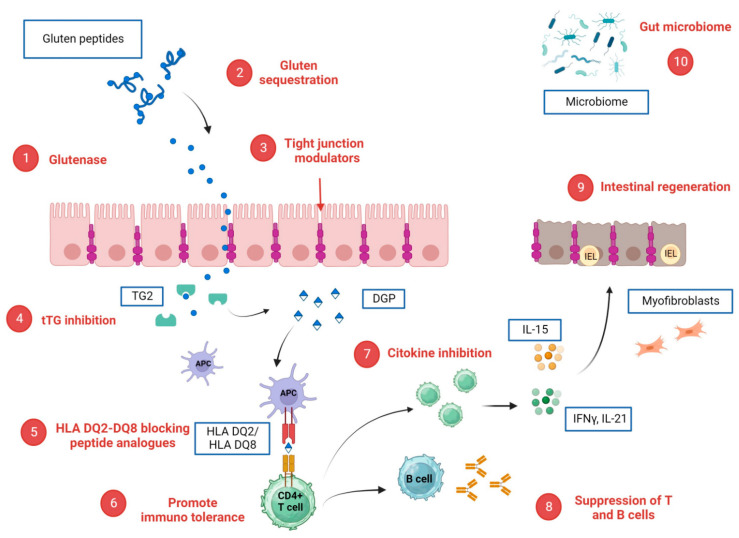
Potential CeD-specific therapeutic targets and corresponding investigational drugs. (**1**) glutenase: latiglutenase, STAN 1, AN-PEP, TAK-062; (**2**) Gluten sequestration: AGY, BL-7010; (**3**) tight junction modulators: larazotide; (**4**) tTG inhibition: ZED 1227; (**5**) HLA DQ2/DQ8 blocking peptide analogues; (**6**) promotion of immuno tolerance: Nexvax2, TIMP-GLIA and KAN 101; (**7**) cytokine inhibition. Anti-IL 15 (AMG714), Tofacitinib, anti CCR9R: CCX282-B; (**8**) suppression of B cells and T cells: rituximab and steroids; (**9**) intestinal regeneration: IMU 856; (**10**) gut microbiome: bifidobacterium longum, lactobacillus strains. Created with BioRender.com.

Glucocorticoids are potent inhibitors of inflammation through the suppression of B cells and of cytokine production by regulatory T cells (Figure 2, point 8) [78]. The use of corticosteroids in CeD has been tested for the treatment of non-responsive celiac disease (NRCD) with a gluten-free diet and represents one of the few treatments available when the non-response is due to refractory celiac disease [79]. Since the use of systemic corticosteroids is limited by systemic side effects, budesonide, a micronized corticosteroid with a high topical effect and low bioavailability, represents an attractive alternative. 

In 2018, a single-center, randomized, open-label trial aimed to evaluate the effects of a short course of prednisolone combined with a GFD on the recovery of celiac disease. Fourteen newly diagnosed CeD children were randomized into a GFD-only group and fourteen into a GFD with prednisolone group (1 mg/kg for four weeks). The use of prednisolone did not lead to a different clinical and serological recovery, but there was a rapid improvement in histological recovery at 8 weeks. However, there was no difference in overall histological improvement at 12 months after starting treatment [80]. Recently, the benefits of a 3-month treatment with budesonide in children with NRCD were observed [81]. Budesonide was also tested to induce clinical response in adults with NRCD, showing a more response in those with diarrhea and less in those with fatigue or other extra intestinal symptoms [82]. The authors also observed that in individuals with RCD, short courses of budesonide were associated with a high risk of clinical recurrence and lack of mucosal recovery, underlying that in these patients, a longer course could be more effective, as shown in Mayo Clinic’s recent series [83]. In this latter retrospective study, budesonide was given 3 times daily as an opened 3 mg enteric-coated capsule. The first and the second daily capsule were opened, placed into apple sauce and swallowed with water; the third daily capsule instead was swallowed intact. With this protocol, they let the drug reach the entire small bowel. The authors observed that budesonide was able to induce clinical and histologic responses in patients affected by RCD I and RCD II, regardless of previous treatment with immunosuppressants (azathioprine) or systemic steroids. Malamut et al. showed complete normalization of the mucosa in only 4 out of 10 patients with RCD-1 taking systemic corticosteroids [84]. A second and less common indication of corticosteroids could be celiac crisis, which is defined as acute onset or rapid progression of gastrointestinal symptoms and requires hospitalization. In 2010, a retrospective study, in which 11 patients with celiac crisis were involved, showed a rapid clinical improvement within 2 weeks in patients who received corticosteroids at dosages of 30 mg, 40 mg or 60 mg [85]. 

Finally, dapsone is the only Food and Drug Administration (FDA)-approved drug used in the treatment of dermatitis herpetiformis and is the drug of choice if gluten-free diet modification is not an option for whatever reason [86,87]. 

#### 6.1.2. tTG Inhibition

Transglutaminase 2 (TG2) is an antigen expressed in the intestinal mucosa, which targets gliadin peptides and deamidates neutral glutamine residues into negatively charged glutamic acid. Due to this modification, deaminated gliadin peptides bind with high-avidity HLA-DQ2 or HLA-DQ8 molecules on mucosal antigen–presenting cells and then activate gluten peptide-specific CD4+ TH1 cells (Figure 2, point 4) [88].

**ZED1227** is an investigational pharmaceutical that is an active-site-directed TG2 inhibitor that prevents the deamidation of gluten and the production of proinflammatory cytokines. ZED1227 was effective and safe in phase 1 clinical trials that involved more than 100 healthy individuals exposed to up to 500 mg (EudraCT numbers, 2014-003044-13 and 2015-005283-42). A phase 2 clinical study compared patients with well-controlled CeD exposed to a placebo to patients who underwent a daily gluten challenge for six weeks with 3 doses of ZED 1227 (10 mg, 50 mg, 100 mg). It assessed that this drug reduced gluten-induced duodenal mucosal injury [89]. Based on these clinical data, a phase 2b “real-life” study started in 2021 that evaluates the efficacy and tolerability of ZED1227 in celiac disease subjects experiencing symptoms despite a gluten-free diet still ongoing (Eudra CT number 2020-004612-97). 

#### 6.1.3. Glutenase

As reported above (Figure 2, point 1), the undigested gliadin peptides are proline-rich and resistant to proteolytic degradation. They can cross tight junctions and reach the lamina propria, activating proinflammatory cascades. Therefore, drugs have been designed to sequester gluten in the intestinal lumen or/and improve its intraluminal digestion. 

**ALV003 (or latiglutinase)** is an investigational pharmaceutical that is an orally administered mixture of two recombinant proteases: ALV001, a cysteine endoprotease B-isoform, and ALV002, a prolyl endopeptidase. In two phase 1 clinical trials, the drug was administered in the fasted state (study 1, *n* = 28) and with a gluten-containing meal (study 2, *n* = 53) and no serious adverse events or reactions were reported at all dose levels of ALV003 (100, 300, 900, and 1800 mg) [90]. 

In two phase 2, double-blind, placebo-controlled clinical trials, well-controlled CeD patients underwent a daily gluten challenge (2 g). Sixteen patients given ALV003 and eighteen given a placebo were evaluated. After 6 weeks of treatment, ALV003 attenuated gluten-induced intestinal mucosal injuries [91]. 

Moreover, another clinical trial assessed the efficacy and safety of latiglutenase in 494 CeD patients. Participants on a GFD for at least 1 year with persistent moderate or severe symptoms and villous atrophy were assigned randomly to groups given a placebo or 100, 300, 450, 600 or 900 mg latiglutenase daily for 12 or 24 weeks. ALV003 did not improve histologic and symptom scores compared to the placebo [92]. However, seropositive patients showed symptoms (abdominal pain and bloating) and QoL benefits from using latiglutenase with meals compared to the placebo [93].

Recently, in a placebo-controlled phase IIb study, in which 21 CeD patients were exposed to 2 gr of gluten per day for six weeks compared to 22 patients exposed to a placebo, Murray et al. reported that latiglutenase (IMGX003) was able to reduce gluten-induced intestinal mucosal damage and symptom severity [94]. In this study, measurements of gluten-immunogenic peptides (GIPs) in urine were used to demonstrate the mechanism of action for this enzyme; indeed, the measurements of GIP in urine indicated 95% gluten degradation in the stomach by latiglutenase. A phase 2 RCT (NCT04243551) is ongoing and aims to evaluate the symptom severity reduction as a primary endpoint in patients with CeD exposed to periodic gluten for 6 weeks. 

**TAK-062** is an investigational pharmaceutical that is an effective endopeptidase, degrading more than 99% of gluten (3 g and 9 g) in vitro within 10 min. In a phase I dose escalation study conducted in healthy participants and CeD patients, Pultz et al. reported that TAK-062 was well tolerated and, for complex meals (1–6 g gluten), median gluten degradation ranging from 97% to more than 99% was found after analyzing the aspiration of stomach contents [95]. A phase 2 RCT (NCT05353985) in patients with active CeD is still ongoing and expected to be completed in May 2025.

**AN-PEP** is a food supplement that is an *Aspergillus niger*-derived endopeptidase that leads to luminal gluten detoxification. Salden et al. and König J et al. have assessed the efficacy of AN-PEP in degrading gluten in the stomach of healthy volunteers or gluten-sensitive subjects [96,97]. The efficacy of this endopeptidase on symptoms was tested in a randomized double-blind placebo-controlled pilot study which showed no symptomatic advantages over the placebo among the 14 involved patients, who had to consume a gluten-containing food product (toast) with AN-PEP for 2 weeks [98]. Furthermore, a phase IV clinical trial has investigated the effect of daily administration of AN-PEP compared to a placebo in terms of the frequency and concentration of GIPs in stool and urine episodes over 4 weeks. Forty CeD patients were enrolled but the results are not yet available (NCT04788797).

#### 6.1.4. Intestinal Regeneration

Once the CD4+ T-cells are activated by antigen-presenting cells, they secrete various cytokines such as IFNγ and IL-21, resulting in an immune cascade that leads to mucosal damage. The mucosal morphological changes in CeD are mainly represented by increased intraepithelial lymphocytes (IELs) with or without villous atrophy of the duodenal mucosa (Figure 2, point 9).

**IMU 856** is an epigenetic regulator that enhances the normal physiological process of gut wall renewal, aiming to restore the villous architecture by regenerative processes of the epithelial lining. IMU 856 acts as a small molecule regulator that stabilizes and enhances the expression of SIRT6 (Sirtuin 6). This protein serves as a transcriptional regulator of the intestinal barrier function and regenerates the bowel epithelium [99]. 

Phase 1/ 1b trials are only published in the Australian trial register, and only one Phase 1b trial was published in May 2023 [99,100,101]. It evaluated patients with well-controlled CeD receiving 28-day treatment of 80 mg or 160 mg of IMU 856 compared to the placebo arm. It was shown that IMU-856 has beneficial effects on symptoms over the placebo, prevents histological damage and enhances nutrient uptake (zinc, vitamin B12). There was also an improvement in citrullin, a biomarker that reflects the health status of enterocytes, and a reduction in IL-2 levels. IMU-856 was safe and well tolerated, and the highest incidence of adverse effects was derived from the high prevalence of ongoing active coeliac disease patients. A phase 2b clinical trial is ongoing in active CeD patients. 

#### 6.1.5. Tight Junction Modulators

Enterocytes are joined together by tight junctions, whose abnormalities underlie the CeD pathogenesis and let indigestible gluten fragments reach the lamina propria and create immunostimulatory epitopes (Figure 2, point 3). **AT 1001**, or larazotide acetate, is an investigational pharmaceutical that is an oral peptide derived from the zonula occludens toxin secreted by Vibrio cholera. Due to the pathogenic role of zonulin-dependent intestinal barrier permeability, AT1001 was tested in celiac patients in different clinical trials (NCT01396213, NCT00620451, NCT00362856, NCT00492960). 

A systematic review, in which only four RCTs (626 patients) met the eligibility criteria (AT-1001, *n* = 465, placebo, *n* = 161), concluded that larazotide acetate is well tolerated and improves symptoms in patients with CeD, particularly those undergoing gluten challenge. On the other hand, the superiority of larazotide acetate over the placebo in reducing intestinal permeability was not reported because the pooled analysis of the change in the urinary LAMA ratio did not significantly differ between AT-1001 and placebo groups [102]. The implications for histologic improvement or immunologic sequelae of larazotide are still unknown. 

An interim analysis led to discontinuation of a recent placebo-controlled Phase III clinical trial which enrolled 525 coeliac patients with persistent symptoms [103]. This study aimed to evaluate the efficacy and safety of larazotide acetate for relieving persistent symptoms in adult patients with coeliac disease on a gluten-free diet. 

#### 6.1.6. Gluten Sequestration

##### Anti-Gluten Antibody

**AGY** is an encapsulated oral egg yolk anti-gliadin polyclonal antibody that neutralizes all CeD-inducing prolamins due to its cross-reactivity (Figure 2, point 2). In the phase 1 open-label trial with a cohort of ten patients on a GFD, AGY improved quality of life, lowered antibodies and lowered LMERs (lactulose mannitol excretion ratios) when taken before meals [104]. A phase 2 RCT that is evaluating the effect of AGY on celiac symptoms is ongoing (NCT 03707730).

##### Polymeric Binders

**BL-7010** is a copolymer poly (hydroxyethyl methacrylate-*co*-styrene sulfonate) (P(HEMA-*co*-SS)) that binds with higher efficacy to gliadin and protects it from enzymatic cleavage by digestive enzymes, preventing the formation of immunogenic peptides. It was shown that BL-7010 was effective at abrogating gluten-associated pathology in gliadin-sensitized NOD-DQ8 mice. Still, it was also correlated with a decrease in TNF-α in response to gliadin in mucosal biopsy specimens of patients with CeD [105,106]. In 2014, a RCT enrolled 40 celiac patients, testing the single and the repeated single administration of these drugs, but the results are still unknown (NCT01990885). 

#### 6.1.7. Cytokine Inhibition

**Tofacitinib** is a pan-JAK inhibitor and is approved to treat inflammatory bowel diseases and rheumatoid arthritis (Figure 2, point 7). Yokoyama S et al. point out the potential value of tofacitinib as a therapy for RCD because they observed a lasting reversal of pathologic manifestations in a transgenic mouse model of CeD treated with tofacitinib [107]. A single report of RCD remission in response to tofacitinib is reported [108]. A phase 2 open-label trial of tofacitinib in type II RCD is ongoing (Eudra CT: 2018-001678-10). 

Another therapeutic target is a CCR9 receptor antagonist. CCR9 is a small intestinal homing receptor that leads to the migration of lymphocytes to the intestine. A phase 2a clinical trial is evaluating the effect of **CCX282-B** on the villous height/crypt depth ratio in CeD patients on a strict GFD compared to a placebo (NCT00540657). Even though the study is completed, the results have yet to be published. Further data are required to determine the safety of these drugs.

#### 6.1.8. Gut Microbiome

Differences in the microbiota have been reported in many inflammatory intestinal diseases and CeD [36] (Figure 2, point 10). The microbiota and their metabolites are thought to play a role in gluten metabolism and, consequently, in CeD onset and severity. They may regulate the permeability of the intestinal barrier and the modulation of the adaptive and innate immune responses [109,110,111]. Recently, a published study has shown that probiotic *Lactobacillus* strains have enzymatic abilities to hydrolyze gluten peptides [112]. Furthermore, distinct *Bifidobacteria* attenuate gliadin-induced immunopathology by producing a serine protease inhibitor (Srp) [113]. A double-blind placebo-controlled study on pediatric patients assessed that *Bifidobacterium longum* CECT 7347 was able to reduce immune markers (serum TNF, IgA in stool and peripheral CD3+ T cells), as also proven in other randomized trials on *Bifidobacterium breve strains* (B632, BR03) [114,115,116,117,118].

A recent meta-analysis performed in 2020 assessed that probiotics could improve gastrointestinal symptoms in CeD patients, while there was insufficient data on the QoL and tumor necrosis factor-a levels [119]. RCTs on the effect of probiotics on mucosal recovery are still lacking, and nowadays, guidelines do not recommend probiotic use in CeD patients. Research in this field is needed in the future.

### 6.2. Injectable Agents

#### 6.2.1. Cytokine Inhibition

Interleukin-15 (IL-15) is highly upregulated in the epithelium and the lamina propria of CeD patients [120]. It can abrogate tolerance to dietary antigens because effector T cells become resistant to inhibition by regulatory T cells [121]. In addition, IL-15 triggers an anti-apoptotic pathway in human intraepithelial lymphocytes [122] (Figure 2, point 7). 

**AMG714** is the first investigated anti-IL-15 monoclonal antibody. In the first phase 2a clinical trial in CeD patients on a GFD, treatments were administered by two subcutaneous injections every 2 weeks for 10 weeks (a total of six doses) and patients without severe villous atrophy at baseline received a gluten challenge (2–4 g daily) during weeks 2–12. It was shown that AMG714 did not prevent mucosal injury due to gluten challenge in comparison to the placebo group at either 150 and 300 mg; however, a smaller increase in IEL was observed at 300 mg and fewer symptoms were observed in the treatment group [123]. Furthermore, in patients with RCD type 2, a phase 2 clinical trial found no difference in the AMG714 group in terms of the reduction in aberrant intraepithelial lymphocytes from baseline [124].

A potential immunomodulator is **Rituximab**, the anti-CD20 antibody. CD20 is a B cell marker, and B cells are involved in the pathogenesis of CeD due to the production of various antibodies (Figure 2, point 8). Two case reports have shown a clinical and biological improvement in CeD in patients treated with Rituximab, but clinical trials are missing [125,126].

#### 6.2.2. Immuno Tolerance Promotion

A T cell-driven adaptive immune response is directed against DGPs. One of the goals of the novel therapeutic development in CeD is to suppress this response (Figure 2, point 6).

The possibility of restoring immune tolerance to gluten in CeD patients has been recently considered. **Nexvax2** is the first peptide-based immunotherapy that aims to suppress or delete disease-causing antigen-specific CD4+ T cells.

In two phase 1 trials, Nexvax2 was administered intradermally, and it has been reported that this drug at doses of 60 µg or higher caused an acute first-dose gastrointestinal reaction (i.e., diarrhea or nausea) and transiently elevated blood concentrations of IL-2 and IL-10. However, with stepwise dose escalation, there were no differences in symptoms between the Nexvax2 group and the placebo [127]. In a phase 2, randomized, double-blind, placebo-controlled clinical study, Nexvax2 was stopped after an interim analysis showed that Nexvax2 did not provide statistically significant protection from gluten-induced symptoms [128].

**TIMP-GLIA (or TAK-101)** is a native gliadin encapsulated in negatively charged poly (dl-lactide-*co*-glycolic acid) nanoparticles. The induction of sustained unresponsiveness to gluten was observed in mouse models with gliadin sensitivity after TIMP-GLIA injection. Inhibition of the proliferation of cytokines IL2, IFNγ, and IL17 and the secretion of gliadin-stimulated T cells were reported [129]. In a phase 1 study, the intravenous administration of TAK-101 was well tolerated, with no profound adverse effects. A change from baseline in circulating gliadin-specific interferon-γ-producing cells at day 6 of gluten challenge in patients with CeD was instead analyzed in a phase 2 study. It included 33 patients who completed the 14-day gluten challenge. TAK-101 induced an 88% reduction in interferon-γ spot-forming units from baseline compared to the placebo. As a secondary endpoint, Vh:Cd deteriorated in the placebo group (−0.63, *p* = 0.002), but not in the TAK-101 group (−0.18, *p* = 0.110) [130].

**KAN 101** is a liver-targeting glycosylation signature conjugated to a deamidated gliadin peptide, which leads to gliadin immuno tolerance. The safety and tolerability of this drug were analyzed recently in the first human phase 1 trial, which was divided into two parts. Part A was an open-label, single-ascending-dose study of intravenous KAN-101 (from 0.15 mg/kg to 1.15 mg/kg). Part B was instead a randomized, placebo-controlled, multiple-ascending-dose study (from 0.15 mg/kg to 0.6 mg/kg), in which patients received three administrations of KAN-101 or a placebo followed by a 3-day oral gluten challenge (9 g per day) 1 week after completing treatment.

During the study, no serious adverse events, dose-limiting toxicities or deaths occurred. It was also reported as a secondary end point that this drug has a rapid systemic clearance and there was not an accumulation on repeated dosing. Further studies are needed to assess the safety and the efficacy of this potential drug [131].

## 7. Conclusions

In the last 20 years, commercial drug development for CeD has accelerated, and includes investigational products repurposed from other diseases as well as novel antigen-specific immunotherapies applicable only to CeD but instructive for other diseases driven by antigen-specific CD4+ T cells. A wide range of drug candidates has been evaluated in preclinical studies and small phase 1/2a trials primarily intended to assess safety, but very few have attracted the substantial investment needed for large phase 2 and phase 3 efficacy studies. The recent FDA statement focuses attention on the unmet needs of adult CeD patients with persistent mucosal injury and symptoms attributed to gluten ingestion. Patient selection and the efficacy of pharmaceutical adjuvants to a GFD would therefore be expected to require gastroenterologists to confirm CeD diagnosis in patients on a GFD and also provide objective evidence of mucosal injury despite adherence to a GFD.

Novel drugs present the potential for non-dietary therapy for CeD that could improve on the efficacy of a GFD in cases where it is not possible to resolve symptoms and gluten damage. In the future, pharmaceuticals could eventually substitute or reduce the rigorous adherence to a GFD now necessary for mucosal recovery.

## Data Availability

Data sharing is not applicable.
